# Acetylation and Deacetylation of DNA Repair Proteins in Cancers

**DOI:** 10.3389/fonc.2020.573502

**Published:** 2020-10-22

**Authors:** Shiqin Li, Bingbing Shi, Xinli Liu, Han-Xiang An

**Affiliations:** Department of Medical Oncology, Xiang'an Hospital of Xiamen University, Xiamen, China

**Keywords:** DNA repair, acetylation, deacetylation, cancer, acetyltransferase, deacetylase

## Abstract

Hundreds of DNA repair proteins coordinate together to remove the diverse damages for ensuring the genomic integrity and stability. The repair system is an extensive network mainly encompassing cell cycle arrest, chromatin remodeling, various repair pathways, and new DNA fragment synthesis. Acetylation on DNA repair proteins is a dynamic epigenetic modification orchestrated by lysine acetyltransferases (HATs) and lysine deacetylases (HDACs), which dramatically affects the protein functions through multiple mechanisms, such as regulation of DNA binding ability, protein activity, post-translational modification (PTM) crosstalk, and protein–protein interaction. Accumulating evidence has indicated that the aberrant acetylation of DNA repair proteins contributes to the dysfunction of DNA repair ability, the pathogenesis and progress of cancer, as well as the chemosensitivity of cancer cells. In the present scenario, targeting epigenetic therapy is being considered as a promising method at par with the conventional cancer therapeutic strategies. This present article provides an overview of the recent progress in the functions and mechanisms of acetylation on DNA repair proteins involved in five major repair pathways, which warrants the possibility of regulating acetylation on repair proteins as a therapeutic target in cancers.

## Introduction

Several mechanistically distinct DNA repair pathways have evolved to restore a myriad of DNA damages induced by exogenous and endogenous stressors, such as radiation, chemical, and biological toxins, as well as reactive oxygen species (ROS) generated during cellular metabolism. Defective DNA repair pathways fail to remove DNA damages, which leads to genomic instability, malignant transformation, and cancer susceptibility. Thus, repressing the activity of DNA repair proteins in cancers might increase sensitivity to other therapeutic regimens (1–3).

Lysine acetylation is one of the most frequently occurring post-translational modifications (PTMs) and has a profound effect in extensive biological processes. Two groups of enzymes, lysine acetyltransferases (HATs) and lysine deacetylases (HDACs), function antagonistically to control the balance between acetylation and deacetylation. Histone protein is the first well-established example of biological functional protein acetylation (4). Acetylation on histones neutralizes the positive charge of the lysine residue, resulting in the de-structure of the chromatin–histone complex. Transcriptional and repair machineries are able to access into the DNA template sites where they modulate the gene expressions or fulfill the damage repair processes (5). Over the past few decades, advances in mass spectrometry allowed the identification of over 2,000 acetylated non-histone proteins participated in widespread cellular processes including DNA repair (6). There is compelling evidence that reversible lysine acetylation is engaged to regulate the functions of repair proteins (6, 7).

Modulating the acetylation of repair proteins has been confirmed as a promising approach for cancer treatment. Alterations in the transcriptional level of various HDACs were observed in numerous cancers, such as colorectal, gastric, esophagus, breast, ovary, lung, pancreas, thyroid, prostate, and oral cancers, as well as malignant melanoma (8). Several HDACIs (belinostat, romidepsin, panobinostat, vorinostat, and chidamide) that obtained the US Food and Drug Administration (FDA) or the China FDA approval have already been applied singly or with other therapeutic drugs in the clinical treatment of various hematologic and few solid tumors. However, the occurrence of high adverse reaction rate and the potential possibility for promoting the pathogenesis or expediting the progression of cancers severely limit the clinical application of HDACI for lacking HDAC subtype specificity (9, 10). Hence, illuminating the acetylation status at specific lysine sites of DNA repair proteins and figuring out the upstream HATs and HDACs as well as downstream substrates are a great help in better understanding the pathogenesis mechanism and developing new targeted therapeutics for clinical treatment of cancers.

## HAT and HDAC in Humans

HATs are a group of enzymes that catalyze the transfer of the acetyl moiety from acetyl-coenzyme A to the lysine residues of the side-chain epsilon amino group. Based on sequence similarities, canonical HATs include three families: GNAT family (GCN5 and PCAF), CBP family (CBP and p300), and MYST family (Tip60, MOZ, MORF, HBO1, and MOF) (11, 12). NAT10, ATAT1, ESCO1, ESCO2, and HAT1 are included as non-canonical HATs (12).

Eighteen kinds of HDACs in eukaryocytes promote the counteraction of lysine acetylation. HDACs are categorized into two major groups: Zn^2+^-dependent HDACs including class I (HDAC1, 2, 3, and 8), II (HDAC4, 5, 7, and 9), IV (HDAC11), and NAD^+^-dependent sirtuin deacetylases including SIRT 1–SIRT7 (6, 12). Although precise functions and substrates of these HATs/HDACs are not thoroughly characterized, acetylation on repair proteins has been implicated in the modulation of DNA repair ability *via* different repair pathways in cancer cells.

## Acetylation on Repair Protein in Major DNA Repair Pathway

Five major pathways including mismatch repair (MMR), base excision repair (BER), nucleotide excision repair (NER), homologous recombination (HR), and NHEJ are devoted to remedying types of DNA lesions (13). The regulation mechanism of acetylation/deacetylation on DNA repair proteins and acetylation-accompanied functions in tumorigenesis and therapeutic response of cancers are summarized in each repair pathway (Table 1).

**Table 1:** HAT and HDAC mediating the acetylation of repair proteins and exact lysine sites.

**Biological process**	**Protein**	**HAT**	**Modified lysine site**	**Function**	**HDAC**	**Modified lysine site**	**Function**
MMR	MSH2	HBO1		DNA MMR activity	HDAC6, HDAC10	845, 847, 871, 892,73	Self-stability/MutSα complex formation
	MLH1	p300	33, 241	MutSα-MutLα complex assemble	HDAC6	33, 241	MutSα-MutLα complex assemble
	MSH6				HDAC1		
BER	TDG	CBP, p300		CBP release/recruit APE			CBP release/recruit APE
	OGG1	p300	338, 341	Self-activity	SIRT3		Mitochondrial genome integrity telomeres stability
	NEIL2	p300	49, 150, 153, 154	Enzymatic activity			
	APE1	p300	6, 7, 27, 31, 32, 35,	Telomeres stability	SIRT1, HDAC1	27, 31, 32, 35	
NER	DDB1	p300, CBP	1,067		SIRT7		Interaction with CULA4
	DDB2		278				
	XPA	CBP	215	Interaction with RPA32	SIRT1, HDAC1	63, 67, 215	Interaction with ATR, RPA, RPA32
	XPG	p300, CBP, PCNA		Chromatin interaction			
HR	ATM	TIP60		Autophosphorylation/self-activity	SIRT7		Deactivation
	NBS1	P300			SIRT1		HR repair deficiency
	RAD51				SIRT1, HDAC4, SIRT2, HDAC2		Expression/HR repair deficiency
	EXO1						
	CtIP				HDAC3		DNA end resection/degradation
	CCAR2	hMOF	112, 215	Protein interaction	SIRT1	112, 215	
	BRG				SIRT1	1,029, 1,033	ATPase activity
	RAD52	p300, CBP		Dissociation from the DSBs	SIRT1, SIRT3		dissociation from the DSBs
NHEJ	DNA-PKcs		3,241, 3,260	Repair efficiency			
	KU70	CBP		DNA binding/repair activity/protein interaction with Bax	SIRT1, HDAC6, SIRT6		Repair capacity/protein interaction with Bax/apoptosis
New DNA fragments synthesis	PARP1	p300, CBP, NAT10	498, 505, 508, 521, 524, 949	Transcriptional coactivator activity/self-activity/ interaction with DNA/stability	SIRT1		Cell death
	RPA1	PCAF and GCN5	163	Interaction with XPA	HDAC6,SIRT1		Interaction with XPA
	PCNA	p300/CBP, Eco1	13, 14, 77, 80, 248, 20	Interaction with DNA Polδ and Polβ/sliding on DNA			
	hSSB1	p300	94	p21 transcription			
	DNA polβ	p300	72	dRP-lyase activity			
	DNA Polι	p300, CBP	550	Unclear			
	FEN1	p300		Self-activity/DNA binding homodimerization/nuclear translocation			
Nucleotide synthesis	RRM2	HAT7	95		SIRT2	95	Homodimerization
Protein synthesis	TyrRS	PCAF	244		SIRT1	244	Nuclear translocation

### Mismatch Repair

**Figure 1:**
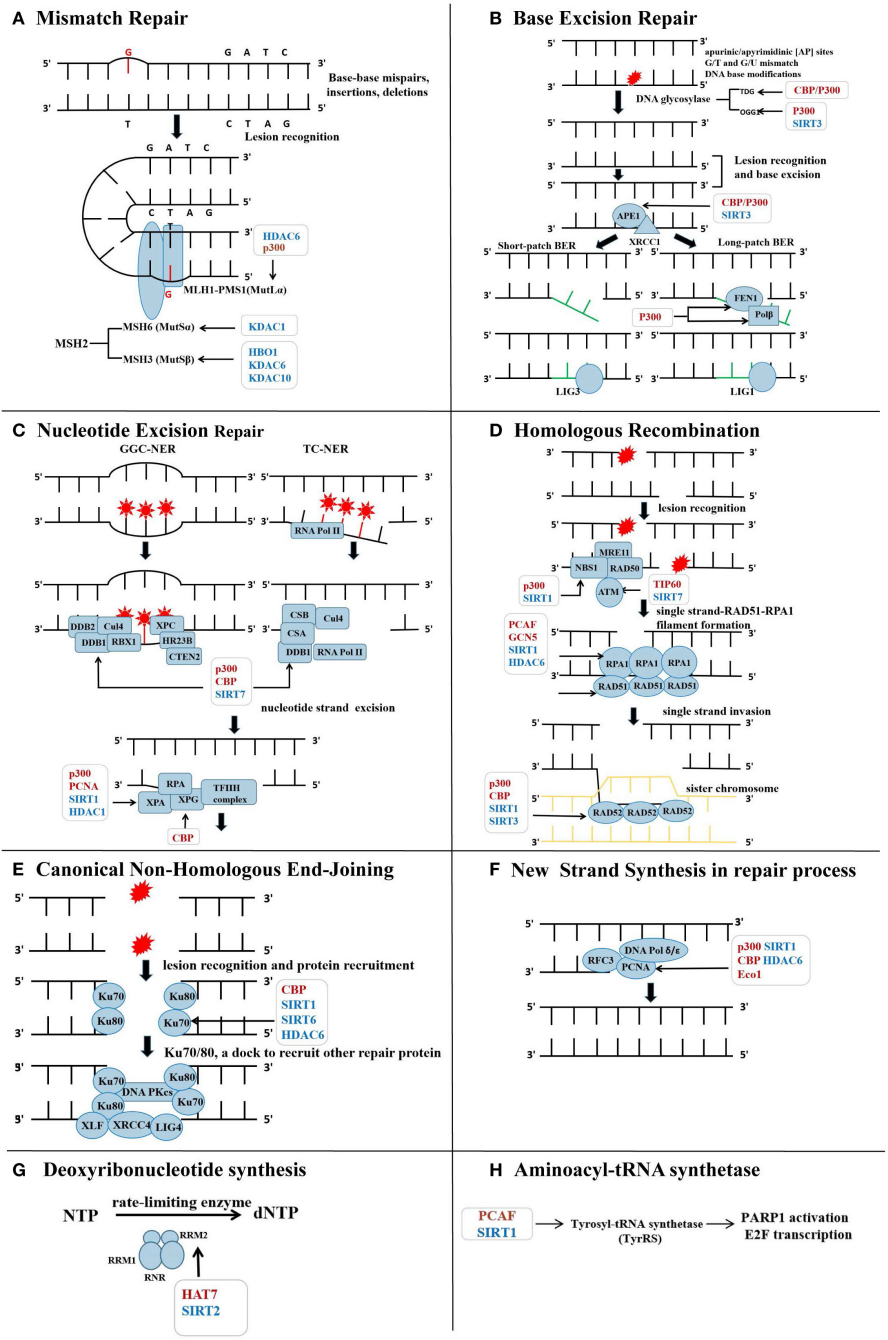
Lysine acetyltransferases (HATs) and lysine deacetylases (HDACs) in the regulation of repair protein across the DNA repair process. **(A)** Acetylation on MutS homolog 2 (MSH2) and MLH1, respectively, affects their assembly with MSH6 or PMS1 into complex, influencing the lesion recognition in mismatch repair (MMR). **(B)** During base excision repair (BER), base excision and new short DNA fragment synthesis are modulated by several HATs and HDACs. Collectively, acetylation on the repair proteins within BER is favorable to BER efficiency. **(C)** Acetylation of DNA-binding protein 1 (DDB1), Xeroderma pigmentosum group A (XPA), and XPG within nucleotide excision repair (NER) is conductive to ensure efficient NER process. Specific HAT and HDAC are highlighted in the figure during the injury recognition and resection process. **(D)** Acetylation on ataxia-telangiectasia-mutated (ATM) help to transmit the double strand breaks (DSBs) damage signals. p300 is recruited to the DSBs sites and acetylates NBS1 in the MRN complex. Acetylation on Recombinant DNA repair protein 51 (RAD51) further promotes homologous recombination (HR). Acetylation on RAD52 acted as a signal to guide its dissociation from the DSBs. **(E)** Ku70 acetylation increases DSBs repair activity and protects the cell from apoptosis. Deacetylated DNA PKcs decreases DSBs repair capacity. **(F)** Acetylation of proliferating cell nuclear antigen (PCNA) promotes its binding to DNA polymerase (DNA Pol) and DNA strands and the formation of new fragments. **(G)** Acetylation on RRM2 disrupts the homodimerization of itself, leading to decline in ribonucleotide reductase (RNR) activity and reduction in deoxynucleotide triphosphate (dNTP) pool. **(H)** Nuclear translocation of tyrosyl necessitates acetylation modification to assist against DNA damage through activating the transcription factor E2F1 and poly (ADP-ribose) polymerase-1 (PARP1) as well as subsequent downstream DNA repair genes.

Base pair mismatch errors that occur during DNA replication are recognized and rectified by the MMR system. Defect of this mutation circumvention system might result in microsatellite instability and increased cancer risks (14). Base–base mispairing and base insertions/deletions are recognized by MutS homolog 2 (MSH2)–MSH6 (MutSα) and MSH2–MSH3 (MutSβ) heterodimeric complexes, respectively (15, 16). Multiple mass spectrum-based studies have identified a diverse number of acetyl-lysine sites on MMR proteins (MSH2, MSH3, MSH6, MLH1, and PMS2) (17, 18). Dysregulation of MSH2 has been pointed out to generate genomic instability, resulting in the development of prostate (19), colorectal (20), and hepatic (21) cancers. A previous study found that the levels of MutSα were controlled through MSH2 acetylation *in vivo*. HDAC6 directly catalyzed the deacetylation of MSH2 at Lys845/847/871/892, which downregulated the stability of MSH2 by promoting ubiquitination and turnover of MSH2. MutSα complex conformation was disrupted, thereby leading to decreased MMR activity. These experiments might provide an interpretation for HDAC6-induced cellular tolerance to DNA-damaging agents (22) (Figure 1A). Depletion of HDAC6 was able to abolish the drugs resistance in non-small cell lung cancer (NSCLC) cells (23), whereas, another study reported that HDAC10 stimulated MMR activity by deacetylation of MSH2 at Lys73. The counteraction of deacetylation on this residue was possibly catalyzed by HAT bound to ORC1 (HBO1) (24). Further studies are necessary to uncover the role of Lys73 deacetylation on MSH2. In addition, an Oncomine database analysis revealed that HDAC10 is probably used as a favorable predictor of prognosis in patients with colon carcinoma (25), which potentially related to increased MMR activity induced by the deacetylation of MSH2.

MLH1-PMS2 (MutLα) heterodimeric complex with a latent endonuclease activity is another indispensable molecule in correcting the mismatched bases. A previous study revealed that p300 and HDAC6 were capable of acetylating and deacetylating MLH1, respectively. The HDAC6 deacetylated MLH1 at Lys33 and Lys241 both *in vivo* and *in vitro*, leading to the disintegration of MutSα-MutLα complex. The DNA damage sensor role of the MutSα-MutLα complex in DNA damage malfunctioned, which impaired MMR activity and induced DNA damage tolerance (26) (Figure 1A). Additionally, proteomics-based substrate trapping identified MSH6 as a substrate of HDAC1 (27). MSH2–MSH6 heterodimeric complex acted as a bidirectional ATPase with ability to change ADP and ATP ratio in DNA damage repair (28). MSH6 mutation might associate with hereditary non-polyposis colorectal and endometrial cancer (29, 30). However, whether acetylation modification on MSH6 interferes with ATPase activity and MMR efficiency still needs further elucidation.

### Base Excision Repair

DNA base lesions, including base losses and bulky modifications, are restored by the BER pathway (31, 32). BER in eukaryotic cell is mainly initiated by 11 kinds of damage-specific DNA glycosylases [uracil DNA glycosylase (UNG), single-strand-selective monofunctional uracil glycosylase 1 (SMUG1), methyl CpG-binding domain protein 4 (MBD4), thymine DNA glycosylase (TDG), 8-OxoG DNA glycosylase (OGG1), MutY glycosylase homolog (MYH), endonuclease III homolog (NTH1), methylpurine DNA glycosylase (MPG), Nei-like DNA Glycosylase 1 (NEIL1), NEIL2, and NEIL1] (33, 34). In addition, AP endonuclease 1 (APE1), ligases (LIG3), X-ray repair cross complementing 3 (XRCC3), and DNA polymerase (DNA Pol) are also required for conducting the following excision–repair process (35). Acetylations on SMUG1, MYH, NTH1, MPG, NEIL1, and NEIL3 were unclear by now, whereas acetylation on UNG (Lys5), MBD4 (Lys232/234/239), and NEIL2 (Lys49/150/153/154) (18, 36) have been identified. To probe into the function of acetylation on these glycosylases may add to an understanding of precisely regulating the BER pathway.

TDG, an enzyme responsible for the removal of thymine moieties from G/T mismatches, generates an abasic site during BER. CBP/p300 was recruited to DNA damage sites by TDG, which potentiated CREB-binding protein (CBP)/p300-dependent transcription and turned into the substrate of CBP/p300. The release of CBP from DNA trinary complexes and the recruitment of APE were both regulated by Ac-TDG, which exhibited a vital role in maintaining genomic stability (37, 38).

Another glycosylase OGG1 is known to remove the 8-oxo-7,8-dihydroguanine generated in DNA oxidative damage. The activity of OGG1 was increased after acetylated by p300 at Lys338 and Lys341, which enhanced the rate of BER *via* reducing affinity for the abasic site under the oxidative stress. Class I HDACs probably catalyzed the reverse deacetylation (39). Another study documented that deacetylation of OGG1 catalyzed by Sirt3 was required in the repair of mtDNA damages induced by oxidative stress (40). Given that oxidative stress and defective mitochondria are a vital driving force for tumorigenesis and tumor development (41, 42) (Figure 1B), the acetylation of OGG1 may be a potential target for cancer therapy.

NEIL2 is charged for repairing oxidative lesions on cytosine and pyrimidine (43). An experimental study conducted in HCT116 cells reported that p300 catalyzed acetylation of NEIL2 at Lys49/150/153/154 in oxidative damage. Moreover, the reversible acetylation of Lys49 was able to decrease both glycosylase and AP lyase activities and regulate the repair activity of NEIL2. In contrast, K153 acetylation was irrelevant to the enzymatic activity (36). Thereby, the acetylation status of essential glycosylases may be considered as an indicator of cancer prognosis.

APE1 is a multifunctional protein involved in BER and the transcription of repair genes. p300, sirtuin (SIRT1), and HDAC1 were reported to mediate the acetylation and deacetylation of APE1, respectively (44, 45). Higher levels of Ac-AP endonuclease 1 (APE1) that exhibited an enhanced DNA repair efficiency for abasic sites have been observed in several primary cancer types, such as colonal, pancreatic, and NSCL cancers, compared with adjacent normal tissues. Acetylation on Lys6/7/27/31/32 of APE1 prevented its degradation and resulted in decreased sensitivity to DNA-damaging agents, which sustained the tumor cell proliferation (46, 47). In addition to increase BER activity, acetylation on APE1 also might impede tumor development through maintaining telomerase stability. Since loss of telomerase stability is a hallmark of cancer cells, exposed telomerase tends to aberrant end-joining reactions, which results in chromosomal fusions and translocations. Human telomerases are guanine-rich regions containing a G-quadruplex structure, which is a hotspot for oxidation forming 8-oxoguanine. As shown by pathogenetic lysine mutation experiments, acetylation at Lys27/31/32/35 on APE1 promoted the cleavage of abasic sites in different G4 structures and maintained the telomerase stability (48) (Figure 1B). Hence, the acetylation of APE1 may underlie a promising target in cancer therapy.

LIG3 is considered as a critical ligase in nuclear and mitochondria BER (49). However, the molecular signaling pathway in regulation of LIG3 acetylation across the process of DNA repair remains to be elucidated. Since LIG3 has been demonstrated as a crucial anti-solid and -hematologic tumor target (50–52), additional works are required to be performed to investigate the connection between LIG3 acetylation and repair efficiency in cancer.

### Nucleotide Excision Repair

Two NER pathways have been described, namely, global genome coupled (GGC)-NER pathway, which handles the removal of helix-distorting DNA lesions throughout the genome, and the transcription coupled (TC)-NER pathway, which deals with transcription-blocking lesions in actively transcribed DNA by RNA polymerase II (RNAP II) (53). GGC-NER is activated by the recognition of damage-induced DNA helix distortions, while the stalling of RNAP II initiates TC-NER at damage sites (54). NER mainly consists of lesion recognition, dual incision on the 3′ and 5′ sides of lesion chains, new DNA fragment synthesis, and gap ligation.

Cullin protein 4 (Cul4)-DNA-binding protein (DDB) complex [RING box-domain protein (RBX1), Cul4, DDB1, and DDB2)] and Xeroderma pigmentosum complementation group C (XPC) complex [(XPC, Rad23 homolog B (HR23B), and Centrin 2 (CETN2)] take charge of damage recognition in GGR-NER. Upon UV light exposure, the DDB complex (DDB1 and DDB2 heterodimer) was recruited to the UV-induced lesions and initiated GGC-NER. DNA repair activity was promoted after the DDB1 subunit combined with p300 and CBP independent of DDB2 *in vivo* (55–57). A proteomic study identified that Lys278 on DDB2 and Lys1067 on DDB1 were possibly acetylated *in vivo* (18). Another study revealed that the interaction between DDB1 and CUL4 was disrupted once DDB1 was deacetylated by SIRT7, which inhibited the activity of the CUL4-ring E3 ubiquitin ligase (CRL4) complex under nucleolar stress induced by actinomycin or 5-fluorouracil. Subsequently, two substrates of CRL4, large tumor suppressor homolog 1 (LATS1) and p73, are accumulated to mediate cell apoptosis (58) (Figure 1C). However, no evidence supported that p300 or CBP mediated the acetylation of DDB1. Although other proteins within Cul4–DDB and XPC complexes all have identified acetyl-lysine sites (59), the corresponding functions of acetylation on these proteins need to be further investigated. Moreover, XPB and XPD subunits in the multisubunit factor Holo–TFIIH complex mainly facilitate DNA duplex unwinding in GGC-NER (60). Whether acetylation plays a role in regulating the functions of XPB and XPD over the repair process also needs to be illuminated.

CSB and Cul4–CSA complex (RBX1, CSA, DDB1) are responsible for damage recognition in TC-NER. XPA interacts directly with all NER core factors at the damage site, aside from XPG, thus, functioning as a scaffold for the excision of the damaged oligonucleotide (61, 62). Following UV-C radiation, XPA was deacetylated by SIRT1 (63). The hypoacetylation state of XPA increased the combination between XPA and RPA, which ensures the following efficient NER. Furthermore, deacetylation on XPA (Lys63/67) by SIRT1 influenced the binding between XPA and RPA32, whereas Lys215 that is located in the ATR-binding region of XPA was deacetylated to enhance TC-NER activity by promoting the bond between ataxia-telangiectasia and Rad3 related (ATR) and XPA (63). By contrast, acetylation of XPA at Lys215 mediated by CBP might play a negative role in regulating XPA–protein interaction, which attenuated TC-NER capacity (64). XPG protein equipped with 3′ endonuclease activity is another core TC-NER factor that is necessary for DNA incision and lesion removal. UV-C irradiation increased the contact between XPG, p300, and CBP, which mediated the acetylation of XPG. Ac-XPG was preferentially released from chromatin (65) (Figure 1C).

### Homologous Recombination

HR mainly restores the lethal DSBs with high fidelity and is usually limited to the S and G2 phases (66). Timely repair of the DSBs restores the structure of DNA double-strand, protectively avoiding large-scale rearrangement of chromosomes (66). Repair proteins involved in the HR process mainly include ATR, ATM, CtIP, cell cycle and apoptosis regulator protein 2 (CCAR2), EXO, MRE11–RAD50–NBS1 (MRN complex), RAD51, RAD52, RPA, DNA Pol, and DNA ligase.

ATR and ATM are two closely related phosphatidylinositol 3′ kinase-like kinases that are activated upon DNA damage, resulting in phosphorylation of various key proteins involved in many kinds of biological processes including cell cycle, DNA repair, and apoptosis (67). Acetylation of ATM has been demonstrated as a critical regulation in self-activation and phosphorylation of various factors in DDR and repair. Acetylation of ATM by Tip60 was a prerequisite for its autophosphorylation and subsequent activation (68). On the other hand, SIRT7 catalyzed deacetylation at K3016 on ATM, promoting the dephosphorylation and deactivation of ATM after repair processes were accomplished (69). However, the effect of the acetylation on ATR was still not investigated.

CtBP-interacting protein (CtIP), exonuclease 1 (EXO1), and MRN complex participate in DNA-end resection and creates stretches of ssDNA coated by replication protein A (RPA) (70). CtIP is contributed to license HR and hamper NHEJ by activating DNA end resection. A study in yeast showed that HDACI valproic acid induced the acetylation and degradation of Sae2 (homologous to CtIP of human) by promoting autophagy (71). Autophagy is performed as a double-edged sword self-degradative system: it participates in the development of MDR and protects cancer cells from chemotherapeutics but can also kill MDR cancer cells escaping from apoptosis pathways. Autophagy induced by anticancer drugs was also able to activate apoptosis signaling pathway in multidrug resistance (MDR) cells, facilitating MDR reversal (72, 73). Hence, a bridge constructed by acetylation of Sae2 and autophagy might be an orientation for the treatment of tumors. The human homologous protein of Sae2, CtIP, is a major player in the choice between NHEJ and HR. At least four acetylation lysine sites were identified on CtIP (74). A previous study has confirmed that the deacetylation of CtIP facilitated DNA end resection (75) HDACI SFN inhibited the activity of HDAC3 in HCT116 cells and promoted the degradation of HDAC3. The interaction between HDAC3 and CtIP was broken due to the turnover of HDAC3, which facilitated the acetylation and degradation of CtIP. HR repair activity was impeded (76). Therefore, acetylation of CtIP perhaps can turn into a potential target for modulating the HR activity in cancer treatment. Moreover, additional studies are required to establish whether acetylation of CtIP has crosstalk with the autophagy pathway.

In a study by using a genome-wide human siRNA library, a CtIP antagonist named CCAR2 was found to inhibit the initiation and limit the extent of DNA end resection, which favored the process of NHEJ (77). Human CCAR2 was discovered as a negative regulator for the deacetylase activity of SIRT1 (78). Acetylation of CCAR2 on Lys54/97 induced by HDACI sulforaphane (SFN) diminished its interactions with HDAC3 and β-catenin. Treatment with the BET (acetyl-lysine reading proteins) inhibitor JQ1 synergized with SFN suppressed tumor development effectively in a preclinical model of colorectal cancer (79). Another study identified that once Lys112 and Lys215 on CCAR2 were acetylated by MYST family HAT hMOF, the interaction of CCAR2–SIRT1 was suppressed, resulting in an increased activity of SIRT1, which further deacetylated CCAR2 to stabilize the SIRT1–CCAR2 complex. Thereby, the function of SIRT1 was limited due to the negative feedback regulation. DNA damage inhibited the binding between hMOF and CCAR2, whereas increased the combination of SIRT1 and CCAR2, which might lead to p53 acetylation and p53-dependent apoptosis (80, 81). Hence, DNA damage-induced acetylation of CCAR2 is critically linked to the p53 apoptosis pathway and might have an effect on the choice of HR and NHEJ in a CtIP-dependent or -independent manner, thereby affecting the therapy response of cancers.

Although an article published in 2009 revealed that EXO1 underwent acetylation modification after treatment with HDACI, the functional alteration of EXO1 induced by acetylation is still unclear (18). In responses to DDR, HAT Tat-interacting protein of 60 kDa (Tip60) was recruited around DNA double-strand breaks (DSBs) to mediate histone acetylation. Other repair factors, such as Rad51 and vital DNA damage sensor MRN complex, were mobilized to anchor on damage sites, forming physical interaction and driving HR (82). Acetylation of ATM catalyzed by Tip60 was critical for its autophosphorylation and activation (68, 83). p300 phosphorylated by ATM interacted with NBS1 and increased its stability, leading to enhanced DNA repair ability (84) (Figure 1D).

Rad51 plays an indispensable role in catalyzing homologous pairing and strand-invasion. A previous study showed that SIRT1 interacted and deacetylated HR repair machinery proteins, including NBS1 and Rad51. Inhibition of SIRT1 impaired HR repair activity, which sensitized the lung cancer cells to WEE1 inhibitor MK-1775-induced DNA damage and apoptosis in lung cancer xenograft model (85). Another HDACI PCI-24781 caused a complete inhibition of RAD51 subnuclear repair foci in response to IR and a significant reduction in the transcription of genes associated with HR, including RAD51. Impaired HR realized a favorable antitumor outcome in combination with poly (ADP-ribose) polymerase (PARP)-inhibitors in HCT116 colon cancer cells (86). In addition, overexpressed HDAC2 and SIRT2 as well as nearly depleted SIRT3 were observed in the aggressive basal-like breast cancer cells. HDAC2 might indirectly influence the expression of RAD51 *via* Mir-182. Experimental study *in vivo* had demonstrated that decreased HR efficiency could lead to breast cancer progression (87) However, whether RAD51 was acetylated by HDAC2 or SIRT2 was not discussed in detail.

Rad52 is a key factor in driving post-invasion steps of both crossover and non-crossover HR pathways. It was recruited to RAD51–ssDNA filament and promoted RAD51 dissociation from DNA (88). Acetylation of RAD52 was regarded as a signal for guiding its dissociation from the DSBs. SIRT1–SIRT3 competed with CBP/P300 to catalyze the deacetylation of RAD52 at the DSBs sites. Accumulated unacetylated RAD52 facilitated the dissociation of RAD51 from the ssDNA filament prematurely, resulting in a reduced ability of HR (88) (Figure 1D).

### Non-homologous End Joining

NHEJ, a non-homologous DSBs repair pathway, including c-NHEJ and alt-NHEJ, undertakes up to 90% of DSBs without sister chromosomes as replication templates. c-NHEJ is carried out mainly by Ku70/Ku80 heterodimer, DNA–PK, LIG4, XRCC4, and XLF, whereas CtIP, PARPs, LIG1/3, and XRCC1 are responsible for the process of the alt-NHEJ pathway (89, 90).

Ku70/Ku80 heterodimer is one of the initial sensors for DSBs and promptly binds to the margin sites in c-NHEJ. Acetylation of Ku70 by CBP *in vivo* disrupted the interaction between Ku70 and apoptotic protein Bax preventing apoptosis. Furthermore, increased acetylation of Ku70 following UV radiation exposure strengthened the combination of Ku70 and DNA, which impaired DSBs repair ability. In contrast, SIRT1 was able to enhance DNA repair capacity *via* catalyzing the deacetylation of Ku70 (91). Reduction of CBP also resulted in an increased DNA repair activity due to decreased acetylation of Ku70 (92). Another study also found that primary green tea polyphenol, epigallocatechin-3-gallate, upregulated the Ku70 acetylation in lung cancer A549 cells. Hyperacetylation of Ku70 blocked the binding between Ku70 and Bax, resulting in the apoptosis of lung cancer cells (93). In neuroblastoma cells, deacetylation of Ku70 by HDAC6 influenced the Ku70–Bax interaction, thus promoting cell death (94, 95). In hepatocellular carcinoma, deacetylation of Ku70 by SIRT6 attenuated Bax-mediated apoptosis (96) (Figure 1E). Collectively, regulating the acetylation of Ku70 will be a promising target for cancer therapy. Together with DNA–PKcs forming DNA–PK complex, Ku70/Ku80 heterodimer served as a docking site for the other c-NHEJ proteins (97, 98). Deacetylation of DNA–PKcs (Lys3241 and Lys3260) *in vivo* has been confirmed to decrease the capacity of DSBs and increase the radiosensitivity of Chinese hamster ovary cells. However, which HAT catalyzed the acetylation of DNA–PKcs needs further validation (99). As a docking site for other c-NHEJ proteins, Ku70/80 heterodimer was known to interact with the LIG4–XRCC4–XLF complex, which plays an essential role in DNA end bridging and ligation (100, 101). However, the influence of acetylation modification on these proteins in c-NHEJ still needs further investigation.

Alt-NHEJ shares similar initiating steps with HR pathways, including the end resection of 5′ ends of the DSBs. PARP1 competed with Ku70 for binding to DNA DSB sites and together with LIG1/3 initiated end-joining in the alt-NHEJ pathway (102). Previous studies discovered that LIG1, LIG3, and PARP1 protein were upregulated in tumorigenic neuroblastoma cells. Inhibition of LIG1 and LIG3 led to DSB accumulation and cell death in neuroblastoma, suggesting the alt-NHEJ pathway as a critical function in cancer cell survival and progression (103) HDACI differentially acetylated DNA repair factors to inhibit NHEJ activity in cancer cells (104). A most recent study demonstrated that the efficiency of DSBs repair by alt-NHEJ and HR, but not c-NHEJ, was increased in mammalian immortalized cells treated with HDACI TSA and PCI-2478. Immunoprecipitation experiments detected that the acetylation levels of c-NHEJ factors (Ku70 and Ku80) and alt-NHEJ factor PARP1 were increased along with a decreased DSBs binding activity. However, no changes in LIG3 and HR factors (Rad51and Rad52) were shown in acetylation levels compared with the untreated group. One explanation for the increased efficiency of HR and alt-NHEJ was that acetylation of PARP1 increased the same end resection in the initial step of these two pathways, while the increased end resection suppressed the c-NHEJ pathway (105). Another study published in 2016 also observed a decreased activity of c-NEHJ with increased acetylation levels of Ku70, Ku80, and PARP1 in acute leukemia cells treated with HDACi. However, vital components in alt-NHEJ including LIG3 and WRN were not affected by HDACI treatment. Instead, the acetylation of PARP1 showed an anomalous persistent binding ability to DNA breaks. Moreover, the activity of alt-NHEJ was not shown to be increased (104). Hence, the molecular mechanism of acetylation on PARP1 in conducting the DNA repair pathway requires deeper investigation (Figure 1E).

PARP1 has been known as a multifaceted and pleiotropic protein across multiple repair pathways. PARPi can cause synthetic lethal effect in BRCA-deficient cancer cells and has already been applied in the clinical therapy of breast, ovarian, prostate, and pancreatic cancers (106–108). PARylation of PARP1 is a prerequisite for contacting XRCC1, Polβ, and LIG3 in damage sites. In addition, PARP1 also facilitated the detection of DNA strand breaks and the choice of repair pathway choice through interaction and modification of DNA repair factors in alt-NHEJ, c-NHEJ, as well as HR (109–115). More than 20 acetylation sites have been identified on PARP1 (18, 116). PTMs including PARylation, acetylation, and ubiquitination exist with mutual competition and crosstalks on PARP1, which is critical for regulating protein functions. Lys498, Lys505, Lys508, Lys521, and Lys524 were related to the regulation of transcriptional coactivator activity of PARP1 (117). Among these lysine sites, Lys498, Lys521, and Lys524 in the automodification domain of PARP1 were targets for PARylation and acetylation at the same time. Dynamic and transient PARylation on PARP1 modulated the recruitment and dissociation of the critical DNA repair proteins in the DNA damage sites (118), whereas acetylation on these lysine sites of PARP1 impeded the access of repair factors (117). In human breast cancer cells, PARP1 was revealed to mediate PARylation of MORC2 in DNA damage sites following DDR (119), which stimulated the ATPase and chromatin remodeling activities of MORC2 and protected the cell from death. MORC2 conversely promoted HAT NAT10-mediated acetylation of PARP1 at Lys949, which blocked the degradation of itself. thereby, the genome repair ability was increased. However, which repair pathway involved in the acetylation of PARP1 was not deeply investigated (120). Moreover, PARP1 also served as a scaffold protein in the damage sites for the functional interaction between BRG (an active subunit of the SWI/SNF chromatin-remodeling complex) and SIRT1. SIRT1 deacetylated BRG at Lys1029 and Lys1033, which stimulated the ATPase activity of BRG, thus remodeling chromatin and promoting HR (121). As described above for the known effects of acetylation on PARP1, it seems that acetylation on different lysine sites plays an opposite role in effecting the efficiency of repair. Hence, precisely regulating the acetylation of lysine sites on these proteins will be more expected.

## Mutual Proteins Throughout Multiple Repair Pathway During DDR

Different repair pathways have an intersection of some proteins, such as ribonucleotide reductase (RNR), tyrosyl-tRNA synthetase (TyrRS), single-stranded DNA binding proteins (SSBs), proliferating cell nuclear antigen (PCNA), DNA and Pol, involved in several mutual biological processes.

### Nucleotide Synthesis

Deoxynucleotide triphosphate (dNTP) metabolism and balance are critical in carcinogenesis. In DNA repair process, the requirement for dNTP was increased since new DNA fragments need to be synthesized. Blocked dNTP synthesis can affect the formation of new DNA fragments and interference DNA repair (122). RNR composed of two large and two small subunits (RRM1 and RRM2) is the rate-limiting enzyme that converts ribonucleotide diphosphates to deoxyribonucleotides (123). In lung cancer H1299 cells and xenografts, SIRT2 specifically deacetylated RRM2 at Lys95 following DDR induced by IR or camptothecin. RNR was activated to ensure sufficient raw materials for DNA synthesis. In contrast, HAT7 induced acetylation of RRM2 at Lys95, which impaired the activity of RNR *via* disrupting RRM2 homodimerization. Consequently, the acetylation of RRM2 at Lys95 suppressed tumor growth both *in vitro* and *in vivo* (124) (Figure 1G).

### Protein Synthesis

TyrRS, one of the 20 aminoacyl-tRNA synthetases, plays a central role in protein synthesis (125). p300/CBP-associated factor (PCAF) and SIRT1 reciprocally regulated the acetylation of TyrRS. Oxidative stress was demonstrated to induce an increased level of PCAF and decreased level of SIRT1, which sequentially led to hyperacetylation at Lys244 on TyrRS. Hyperacetylated TyrRS was promoted to translocate into the nucleus where it protected against DNA damage by activating the transcription factor E2F1 and PARP1 as well as subsequent downstream DNA repair genes. The activity of hyperacetylated TyrRS itself was decreased (126). In addition, acetylation of multiple aminoacyl-tRNAs has been reported in the proteomic data (127) (Figure 1H). The significance of acetylation on these tRNA synthetases remains to be elucidated.

### New DNA Fragments Synthesis

Several DNA damage events generate the exposure of ssDNA. Naked ssDNA is susceptible to suffer further damage induced by various physical and chemical factors. Hence, before repair machinery assembled onto the damage sites, ssDNA needed to be protected by the SSB protein family (128). In humans, four kinds of SSB proteins have been identified, namely, mitochondrial SSB (mtSSB), hSSB1, hSSB2, and RPA (129). Evidence indicated that acetylation of RPA1 (Lys163) catalyzed by PCAF and GCN5 enhanced the interaction between XPA and RPA1 after UV-damage, leading to retention of XPA and eventual efficient repair, whereas HDAC6 and SIRT1 removed this acetyl group from RPA1 after the repair process was accomplished. In addition, UV-induced acetylation of RPA1 was also critical for efficient removal of CPDs and 6-4PPs (130, 131) (Figure 1D). hSSB1 has been implicated in BER (132), HR (128), and NHEJ (128). The combination between hSSB and p300 was required for efficient transcriptional activation of the p53 target gene p21 in p53 wild-type cancer cell lines after exposure to IR (133). Another study uncovered that K94 acetylation on hSSB1, which was mediated by p300, SIRT4, and HDAC10, impaired ubiquitin-mediated degradation by proteasomes. Moreover, hSSB1-K94R mutant had reduced cell survival in response to DNA damage induced by radiation or chemotherapy drugs. C646, an inhibitor of the p300/CBP, significantly enhanced the chemosensitivity of cancer cells to etoposide, adriamycin, and camptothecin. A positive correlation between the level of hSSB1 and p300 was also observed in clinical colorectal cancer samples with immunohistochemistry (134). As for mtSSB and hSSB2, further investigations are needed in understanding the role of acetylation modification on them in the DNA repair process and cancers.

PCNA acts as a master coordinator responsible for timely recruiting DNA replication and repair factors to accurately erase DNA lesions (135). A study showed that following UV irradiation, P300 and CBP attached to the C-terminal domain of PCNA and acetylated PCNA at Lys13, Lys14, Lys77, Lys80, and Lys248 in human embryonic fibroblasts and HeLa cells. The interaction between ac-PCNA, DNA pol δ, and pol β was enhanced. As there was inhibited ubiquitination and consequent degradation of PCNA, DNA synthesis activity and repair efficiency were increased. Mutation of these lysine sites increased the cell sensitivity to UV irradiation (18, 136). Except for acetylation-related self-degradation, another study complemented that in the S-phase, yeast cells, exposed to the DNA-damaging agent, Lys20 acetylation on PCNA catalyzed by HAT Eco1 (homologous to ESCO1 in human), regulated its sliding on DNA strands, thereby, favoring sister-chromatid cohesion in HR (137) (Figure 1F). Whether this regulatory mechanism exists in mammals needs further investigation.

DNA Pol catalyzes the polymerization of deoxynucleotides during DNA replication and DNA repair, which ensures the maintenance of the genetic information and faithful transmission through generations. In total, there are four major families (A, B, X, and Y family) of polymerases including 15 human polymerases that work in the five major pathways and in other three special repair pathways including trans-lesion DNA synthesis, DNA interstrand crosslink repair, and V(D)J recombination (138). Dysregulated expression and mutation of polymerases have been linked with the pathogenesis of cancers (139, 140). In 2002, Hasan et al. discovered that DNA Polβ in the X family formed a complex with the p300 and was acetylated at Lys72 both *in vitro* and *in vivo*. The dRP-lyase activity of Polβ was impaired along with a severely reduced BER (141). Moreover, Polβ catalyzed the formation of a 5′ flap from the 3′ incision during the long-patch BER pathway. Finally, FEN1 was responsible for incising the 5′ flap to complete the repair process (142). Inhibiting the expression of FEN1 restrained the progression and increased the chemo-sensitivity of breast cancer cells (143) (Figure 1B). Acetylation of FEN1 by p300 reduced the DNA binding activity and inhibited its endonuclease activity, which impaired flap cleavage and repair ability (144, 145). A recent study in 2019 pointed out that Polι of the Y family, a non-canonical polymerase involved in trans-lesion DNA synthesis, was acetylated at Lys550 by p300/CBP in response to SN2 alkylating agents (methyl methanesulfonate and dimethyl sulfate) (146). Further studies are needed to clarify the role of acetylation on Polι and other DNA Pols in DNA repair.

## Summary

The dysfunction of repair proteins caused by aberrant acetylation modification is strongly connected with pathogenesis, development, and drug resistance of cancers. Abnormal expressions of HDACs have been detected in a variety of tumors. Based on the favorable therapeutic response in clinical trials, several HDACIs have been approved in the treatment of hematologic tumors and a few solid tumors. However, the alteration of acetylation on repair proteins in many types of solid tumors is less focused, and the affected downstream signaling pathways are also unclear. Digging deeper into the regulations of acetylation on DNA repair proteins may shed light on the pathogenesis of tumors and contribute to the discovery of new drugs targeting this PTM for better therapeutic effects of cancers.

## Author Contributions

SL collected the published data, wrote the paper, as well as made the figures. BS and XL participated in the discussion of the content of the review. H-XA conceived and revised this review. All authors contributed to the article and approved the submitted version.

## Conflict of Interest

The authors declare that the research was conducted in the absence of any commercial or financial relationships that could be construed as a potential conflict of interest.

## Figures and Tables

**Table:** 

ALL	acute lymphocytic leukemia	FL	follicular lymphoma
alt-NHEJ	alternative non-homologous end joining	MM	multiple myeloma
AML	acute myelocytic leukemia	MMR	mismatch repair
APE1	AP endonuclease 1	MOF	males-absent-on-the-first
ATM	ataxia-telangiectasia-mutated	MOF	KAT8, Lysine Acetyltransferase 8
ATR	ataxia-telangiectasia and rad3 related	MORC2	Microrchidia family CW-type zinc finger 2
BER	base excision repair	MORF	MOZ-related factor
BET	bromodomain and extra terminal domain	MOZ	Monocytic leukemia zinc-finger protein
BRCA	BRCA1 DNA repair associated	MPG	Methylpurine DNA glycosylase
BRG	ATP-dependent helicase SMARCA4	MSH2	MutS homolog 2
CBP	CREB-binding protein	mtDNA	mitochondrial DNA
CCAR2	cell cycle and apoptosis regulator protein 2	mtSSB	mitochondrial SSB
CETN2	centrin 2	MYH	MutY glycosylase homolog
c-NHEJ	canonical non-homologous end-joining factor	NEIL1	Nei Like DNA Glycosylase 1
CRL4	CUL4-ring E3 ubiquitin ligase	NER	Nucleotide excision repair
CSA	Cockayne-syndrome A	Non-HL	non-Hodgkin Lymphoma
CTCL	cutaneous T-cell lymphoma	NSCLC	Non-small cell lung cancer
CtIP	CtBP-interacting protein	NTH1	endonuclease III homolog
Cul4	cullin protein 4	OGG1	8-OxoG DNA glycosylase
DDB1	DNA-binding protein 1	PARP-1	poly (ADP-ribose) polymerase-1
DDB2	DNA-binding protein 2	PARPi	PARP-1 inhibitor
DDR	DNA damage response	PARylation	poly-ADP-ribosylation
DLBCL	diffuse large B-cell lymphoma	PCAF	p300/CBP-associated factor
DNA pol	DNA polymerase	PCNA	proliferating cell nuclear antigen
DNA-PK	DNA-dependent protein kinase	PTCL	peripheral T cell lymphoma
DNA-PKcs	DNA-dependent protein kinase catalytic subunit	PTM	post-translational modification
dRP	deoxyribose phosphate	RAD51	recombinant DNA repair protein 51
dNTP	deoxynucleotide triphosphate	RAD52	Rad52 homolog
DSBs	Double strand breaks	RBX1	RING box-domain protein
ER	estrogen receptor	RNAP II	RNA polymerase II
ESCO1	establishment of sister chromatid cohesion n-acetyltransferase 1	RNR	ribonucleotide reductase
EXO	exonuclease 1	ROS	reactive oxygen species
FDA	Food and Drug Administration	RPA	replication protein A
FEN1	flap structure-specific endonuclease 1	SAHA	suberoylanilide hydroxamic acid
FL	follicular lymphoma	SFN	Sulforaphane
GCN5	general control non-derepressible 5	SIRT	Sirtuin
GGC-NE	global genome coupled-NER	SMUG1	single-strand-selective monofunctional uracil glycosylase 1
GNAT	Gcn5-related N-acetyltransferase	SSBs	single-stranded DNA binding proteins
HAT1	lysine acetyltransferase 1	SWI/SNF	switching/sucrose non-fermenting
HATs	lysine acetyltransferases	ATAT1	α-tubulin N-acetyltransferase 1
HBO1	HAT bound to ORC1	TC-NER	transcription coupled-NER
HDACI	HDAC inhibitor	TDG	thymine DNA glycosylase
HDACS	lysine deacetylases	Tip60	Tat-interacting protein of 60 kDa
HER2	human epidermal growth factor receptor-2	TyrRS	tyrosyl-tRNA synthetase
HL	Hodgkin lymphoma	UNG	Uracil DNA glycosylase
HR	homologous recombination	WEE1	Wee1-like protein kinase
HR23B	Rad23 homolog B	XLF	XRCC4-like factor
IR	ionizing radiation	XPA	Xeroderma pigmentosum group A
LATS1	large tumor suppressor homolog 1	XPB	Xeroderma pigmentosum complementation group B
LIG3	Ligases	XPC	Xeroderma pigmentosum complementation group C
LIG4	DNA ligase IV	XPD	Xeroderma pigmentosum complementary group D
MBD4	methyl CpG binding domain protein 4	XPG	Xeroderma pigmentosum complementation group G
MDR	multidrug resistance	XRCC3	X-ray repair cross complementing 3
MDS	myelodysplastic syndrome	XRCC4	X-ray repair cross complementing 4
